# Construction of a hierarchically porous A–π–A conjugated porous polymer (CPP)/Bi nanoparticle heterojunction with enhanced photocatalytic performance

**DOI:** 10.1039/d6ra03144g

**Published:** 2026-07-06

**Authors:** Shunzhong Gong, Shan Jiang, Kong Liu, Jin Bai, Rao Tao, Yepeng Yang, Shulin Gao, Yeit Haan Teow, Haidong Ju

**Affiliations:** a Yunnan Key Laboratory of Metal-Organic Molecular Materials and Device, Yunnan Engineering Technology Research Center for Plastic Films, School of Chemistry and Chemical Engineering, Kunming University Kunming 650214 China skyleo2003@126.com hdju1977@outlook.com; b Department of Chemical and Process Engineering, Faculty of Engineering & Built Environment, Universiti Kebangsaan Malaysia 43600 UKM Bangi Selangor Darul Ehsan Malaysia

## Abstract

Bismuth (Bi), with its abundant reserves, holds great potential as a plasmonic-enhancing component in composite photocatalysts. A benzothiadiazole-triazine-functionalized two-dimensional olefin-linked (vinylene-linked) CPP enhanced by plasmic Bi nanoparticles (NPs) was synthesized. Localized surface plasmon resonance (LSPR)-assisted A–π–A type conjugated polymer (CPP) photocatalysts exhibit significant advantages in energy band design, showcasing their uniqueness in conjugated units and pore size. The benzothiadiazole-triazine olefin-linked CPP possessed an A–π–A characteristic structure, along with hierarchical porous characteristics, and a specific surface area of 569.16 m^2^ g^−1^. In this study, TMTBT-CPP was synthesized using 2,4,6-trimethyl-1,3,5-triazine (TMT) and 4,4′-(benzothiadiazole-4,7-diyl)dibenzaldehyde (BT) as building blocks, and Bi was grown *in situ* on the material surface to form a plasmonic-CPP heterostructure. This heterostructure enhanced visible-light harvesting and accelerated electron transfer. Results showed that the photodegradation efficiency of Bi/TMTBT-1 (Bi : TMTBT = 1 : 1) was 85.7% for degrading 50 mL of rhodamine B solution for degrading 50 mL rhodamine B solution with an initial concentration of 20 mg L^−1^ over 1 h, and its catalytic activity was significantly superior to that of TMTBT alone.

## Abstract 2

Hierarchical porous A–π–A TMTBT-CPP was synthesized, and plasmonic Bi nanoparticles were loaded *in situ* to fabricate Bi/TMTBT plasmonic heterostructures. The LSPR of Bi suppresses carrier recombination, and Bi/TMTBT-1 delivers superior visible-light RhB degradation performance over pure TMTBT-CPP.

## Graphical abstract

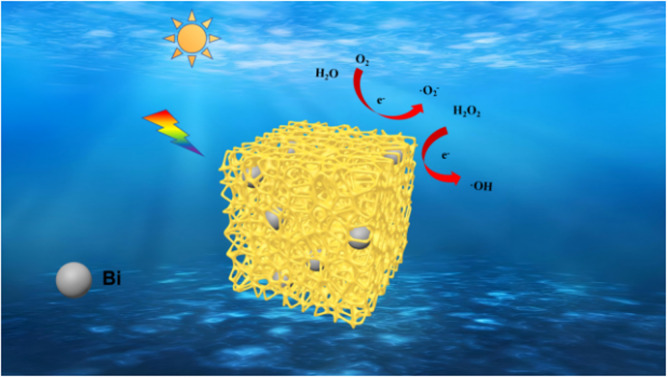

## 1 Introduction

Photoinduced electron transfer (PET), a core photochemical process analogous to natural photosynthesis, realizes photon-exciton conversion and subsequent charge separation, which underpins various applications including photocatalysis,^1,2^ photoelectrochemical energy storage,^3^ and photoinitiated polymerization.^4^ Driven by the increasing demand for sustainable energy and environmental remediation, the rational design of advanced photoactive materials has become a key research direction in photocatalysis.

The introduction of electron donor/acceptor (D/A) units and the construction of built-in electric fields are well-established strategies to accelerate photogenerated carrier separation and improve photocatalytic efficiency.^5^ Although small molecules and supramolecular assemblies, regulated by electronic push–pull effects, are promising for environmental remediation and energy conversion, their practical applications are limited by inferior stability and recyclability. Conjugated polymers possess structural tunability, low cost and favorable processability, outperforming conventional inorganic semiconductors. Nevertheless, severe charge recombination and inadequate light absorption still limit their photocatalytic activity. As outstanding organic semiconductors, conjugated porous polymers (CPPs) have emerged as promising photocatalytic materials owing to their high porosity, large specific surface area and tunable electronic structures, which provide abundant active sites and promote mass transport.^6–8^

With the rapid development of bottom-up approaches for organic conjugated semiconductors, the design of π-conjugated materials with efficient electron delocalization and favorable conductivity has become a research hotspot. Traditional imine-linked C=N porous materials realize photogenerated carrier separation relying on the types and orientations of linkage bonds.^9^ Adjusting the D/A molar ratio of polymer backbones can effectively optimize the separation and migration of photogenerated charges, thereby enhancing the photocatalytic hydrogen evolution activity.^10^ Benefiting from the rational design of donor–acceptor structures, D–A conjugated porous polymers (D–A CPPs) integrate the structural diversity and excellent semiconductor characteristics of CPPs,^11^ and have been extensively applied in organic catalytic transformations^12^ and fluorescent chemical sensing.^13^ Moreover, the semiconductor performances and microscopic photoelectron-transfer behaviors of CPPs can be precisely modulated *via* D–A structural engineering.^14–16^ Based on the above advantages, emerging CPPs with extensive π-conjugated skeletons possess versatile photophysical and electronic properties, showing great potential for light harvesting and high-efficiency photocatalysis.

Nevertheless, the fabrication of CPPs with high photocatalytic activity, thermal stability and cycling durability remains a major challenge for visible-light photocatalysis. Typical p-type CPPs are constructed with electron-deficient units, such as triazine and benzothiadiazole, which possess low LUMO levels to promote hole migration and oxygen evolution.^9,17,18^ In contrast, n-type CPPs adopt electron-rich monomers including carbazole, pyrene and porphyrin with high-lying HOMO levels, utilizing electrons as dominant charge carriers. Rational selection and assembly of conjugated building blocks is therefore essential to fabricate high-performance CPP photocatalysts. Heterojunction construction with plasmonic metals has proven effective for narrowing band gaps, broadening visible-light absorption and prolonging carrier lifetime, thus facilitating charge separation.^19^ Meanwhile, extended π-conjugation further optimizes electron delocalization and suppresses photogenerated charge recombination.^20^ Among various plasmonic candidates, bismuth (Bi) is a low-cost and earth-abundant non-noble metal.^21^ Its unique surface plasmon resonance (SPR) effect can significantly enhance light harvesting, inhibit electron–hole recombination and improve photocatalytic activity, exhibiting good compatibility with conventional semiconductor photocatalysts, such as TiO_2_, g-C_3_N_4_ and BiVO_4_.^22–24^

**Scheme 1:**
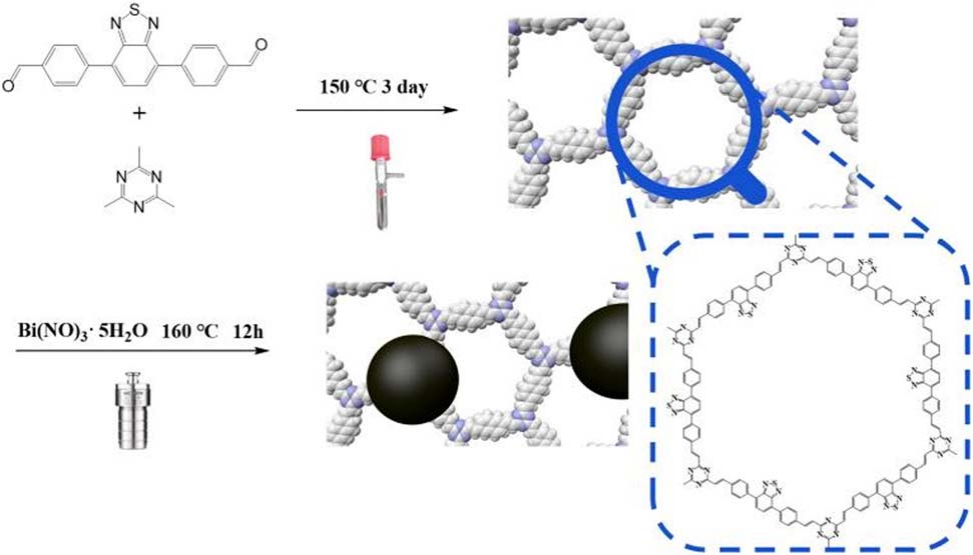
Preparation of Bi/TMTBT.

Notably, vinylene-linked TMTBT porous materials, synthesized *via* aldol condensation, feature robust irreversible C=C bonds and electron-withdrawing skeletons, which endow the materials with superior structural and thermal stability.^25^ Unlike conventional triazine/benzothiadiazole-based CPPs and COFs with reversible imine linkages, this A–π–A conjugated framework also enables efficient intramolecular charge transfer. Benefiting from the large specific surface area and high thermal stability (510 °C) of TMTBT-CPP, Bi nanoparticles can be uniformly immobilized onto the polymer network. The synergistic effect between the SPR property of Bi and the intrinsic advantages of TMTBT-CPP effectively suppresses charge recombination and enhances photocatalytic degradation activity. Herein, we successfully prepared vinylene-linked TMTBT-CPP and fabricated a Bi nanoparticle-modified plasmonic heterojunctions *via* an *in situ* route (as shown in Scheme 1). The morphology, structure and photocatalytic performance of the as-obtained samples were systematically investigated. Rhodamine B (RhB) was selected as a model organic pollutant to evaluate the visible-light photocatalytic degradation performance. Finally, a reasonable photocatalytic mechanism of the Bi/TMTBT plasmonic heterojunction was proposed and elucidated.

## 2 Experimental section

### 2.1 Materials

2,4,6-Trimethyl-1,3,5-triazine (TMT, 99%), 4,4′-(benzo[c][1,2,5]thiadiazole-4,7-diyl)dibenzaldehyde (BT, 98%), benzoic anhydride (98%), bismuth nitrate pentahydrate (>99%), *N*,*N*-dimethylacetamide (>99%), trifluoroacetic acid (>99%), ethanol (>99.7%), ethylene glycol, methyl alcohol, and tetrahydrofuran were purchased from Aladdin-Reagents Ltd (Shanghai, China) and used without further purification.

### 2.2 Preparation of TMT-BT CPP

The synthesis of TMT-BT CPP was slightly modified from the literature. It involves the condensation of 2,4,6-trimethyl-1,3,5-triazine (TMT, 23 mg, 0.18 mmol) and 4,4′-(benzo[*c*][1,2,5]thiadiazole-4,7-diyl) dibenzaldehyde (BT, 96 mg, 0.28 mmol) under the catalysis of trifluoroacetic acid. The reactants were dissolved in a mixture of 3 mL of ortho-dichlorobenzene, 1 mL of *N*,*N*-dimethylacetamide, 50 mg of benzoic anhydride, and 1 mL of trifluoroacetic acid, followed by ultrasonic treatment for 15 min to achieve a homogeneous mixture. The mixture was then transferred to a glass tube, followed by three liquid-nitrogen freeze-degassing cycles, and reacted at 150 °C under vacuum conditions for 72 hours. Subsequently, it was subjected to vacuum drying at 60 °C for 10 hours to obtain an orange powder of TMTBT.

### 2.3 Preparation of Bi/TMTBT

Bi was grown *in situ* on the surfaces of CPP using a one-step solvothermal reduction method. Under magnetic stirring, a specific quantity of Bi(NO_3_)_3_·5H_2_O was dissolved in 10 mL of ethylene glycol. Subsequently, 50 mg of the previously prepared CPP was added, and the mixture was stirred for one hour. The resultant suspension was transferred into a polytetrafluoroethylene (PTFE)-lined stainless steel autoclave and heated at 160 °C for 12 hours.^26^ The resulting powder was meticulously washed with deionized water and anhydrous ethanol, followed by vacuum drying at 60 °C for 12 hours to obtain Bi/TMTBT. Various Bi(NO_3_)_3_·5H_2_O to TMTBT mass ratios (0.5, 1, and 1.5) were employed to prepare Bi/TMTBT-*x* (Bi/TMTBT-0.5, Bi/TMTBT-1, and Bi/TMTBT-1.5).

### 2.4 Characterization

X-ray diffraction (XRD) was carried out using a Bruker D8 Discover instrument with a Cu K*α* radiation wavelength of 1.54060 Å. Fourier-transform infrared (FT-IR) spectroscopy was carried out using a Cary 640 spectrometer to analyze the surface functional groups of the samples. Scanning electron microscopy (SEM) was carried out using a ZEISS Sigma 300 microscope to observe the surface morphology of the samples. Transmission electron microscopy (TEM) was carried out using a JEM-2100Plus microscope (JEOL, Japan) to characterize the microscopic microstructure of the samples. X-ray photoelectron spectroscopy (XPS) was carried out using a Thermo Scientific K-Alpha system to investigate the surface elemental composition and chemical states. Diffuse reflectance ultraviolet–visible (DR UV–Vis) spectroscopy was carried out using a Shimadzu UV-3600i Plus spectrophotometer to test the optical absorption properties. Electrochemical impedance spectroscopy (EIS) and transient photocurrent response measurements were carried out using an electrochemical workstation (CS2350H, Wuhan Corrtest Instrument Co., Ltd, China). Electron paramagnetic resonance (EPR) measurements were carried out using an EPR spectrometer (EPR200-Plus, NQI Quantum Technology Co., Ltd, China) to detect active radical species. The photocatalytic performance of the samples was evaluated by the photodegradation of RhB aqueous solution under a 45 W incandescent lamp. Typically, 5 mg of the prepared catalyst was uniformly dispersed in 50 mL of RhB solution with an initial concentration of 20 mg L^−1^, and the photodegradation reaction was performed for 1 h under continuous illumination.

## 3 Results and discussion

**Fig. 1:**
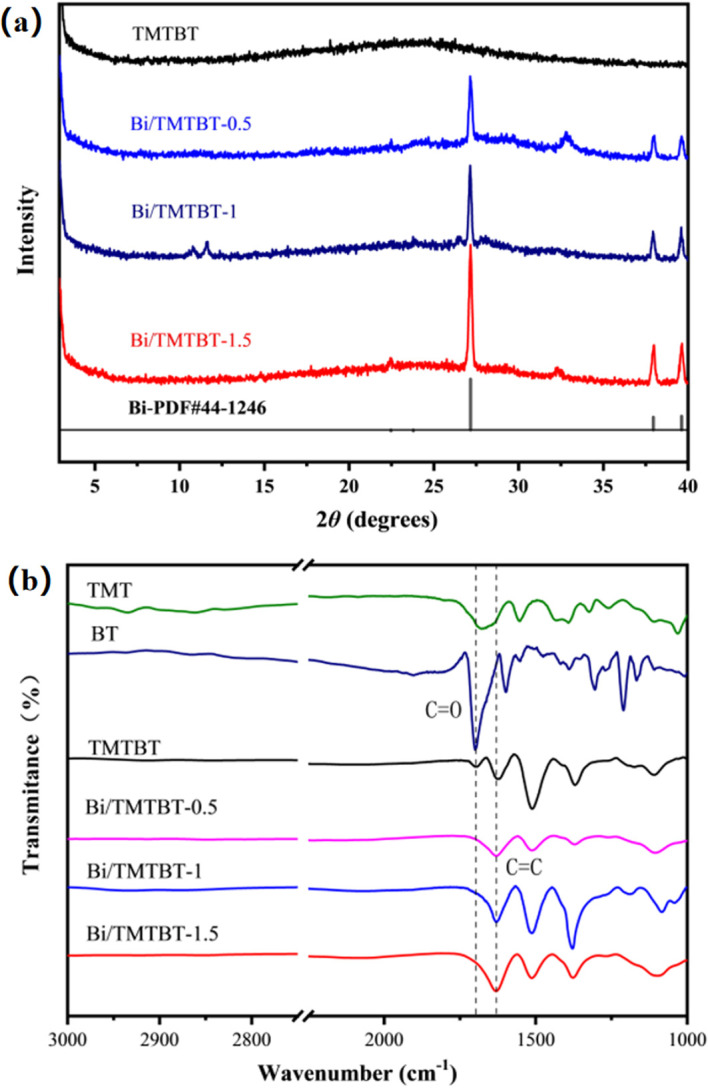
(a) XRD patterns of TMTBT and all the Bi/TMTBT samples. (b) FT-IR spectra of all the as-prepared samples.

X-ray diffraction (XRD) analysis was carried out to investigate the phase composition and crystal structure. All the XRD data for the various composite ratios of Bi/TMTBT-*x* are displayed in Fig. 1a. In Fig. 1a, diffraction peaks corresponding to Bi (JCPDS card number 44-1246) at 27.2°, 37.9°, and 39.6° attributed to the (012), (104), and (110) planes, respectively, are clearly visible, indicating the successful loading of Bi onto TMTBT. Moreover, as the proportion of Bi increases, the intensity of the diffraction peaks also increases.

Furthermore, the Fourier-transform infrared (FT-IR) spectra of all the samples are presented in Fig. 1b. The raw material BT exhibits an infrared absorption peak at 1698 cm^−1^, corresponding to C=O. After synthesizing TMTBT, the intensity of the C=O absorption peak significantly decreases, and a new absorption peak at 1630 cm^−1^ emerges, indicating the formation of C=C, thus confirming the successful synthesis of the polymer. Upon further compositing with Bi, the C=O peak completely disappears and transforms into a peak corresponding to C=C, suggesting that the process of Bi compositing promotes the synthesis of TMTBT.

**Fig. 2:**
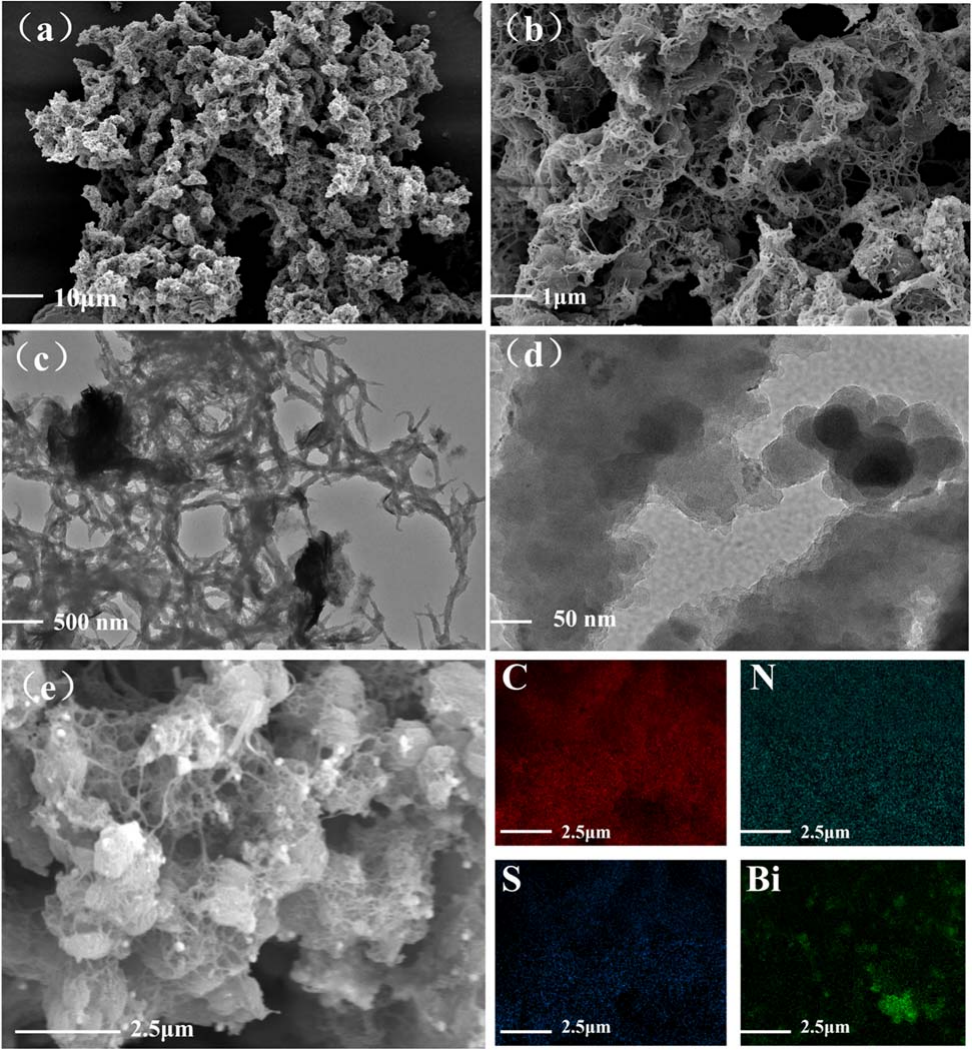
SEM images of (a and b) Bi/TMTBT-1, TEM images of (c and d) Bi/TMTBT-1, and (e) EDS mapping of the Bi/TMTBT-1 composite.

Fig. 2a and b presents scanning electron microscopy (SEM) images revealing that TMTBT possesses a network structure composed of intertwined nanowires. Additionally, it features a rich macroporous structure. Meanwhile, Bi nanoparticles with sizes of around 100 nm are formed *via in situ* growth on the material surface, and the pore structure of the host is well preserved. TEM characterization reveals that the sample has a network structure composed of intertwined nanowires (Fig. 2c and d). Unfortunately, the indistinct contrast between the CPP phase and Bi nanoparticles prevented us from capturing enough Bi particles to conduct a valid size-distribution analysis. Furthermore, elemental mapping analysis confirms the uniform distribution of carbon (C), nitrogen (N), sulfur (S), and bismuth (Bi) elements in Bi/TMTBT-1 (Fig. 2e). It has been documented that the uniform distribution of metal nanoparticles can induce plasmonic metal light-scattering effects, thereby increasing the photon path length within the semiconductor. This, in turn, enhances light absorption and charge separation.^27^ The growth of Bi nanoparticles on TMTBT-CPP is mainly attributed to abundant anchoring sites on the polymer skeleton. The triazine ring provides lone-pair electron-rich nitrogen sites for strong N–Bi coordination interaction, while the benzothiadiazole unit contains electronegative N and S heteroatoms that effectively adsorb and anchor Bi species during *in situ* growth. In addition, the fully conjugated π-electron framework of TMTBT-CPP further strengthens the interfacial bonding *via* metal–π interaction, enabling the uniform and stable immobilization of Bi nanoparticles and the construction of intimate heterojunction interfaces.

**Fig. 3:**
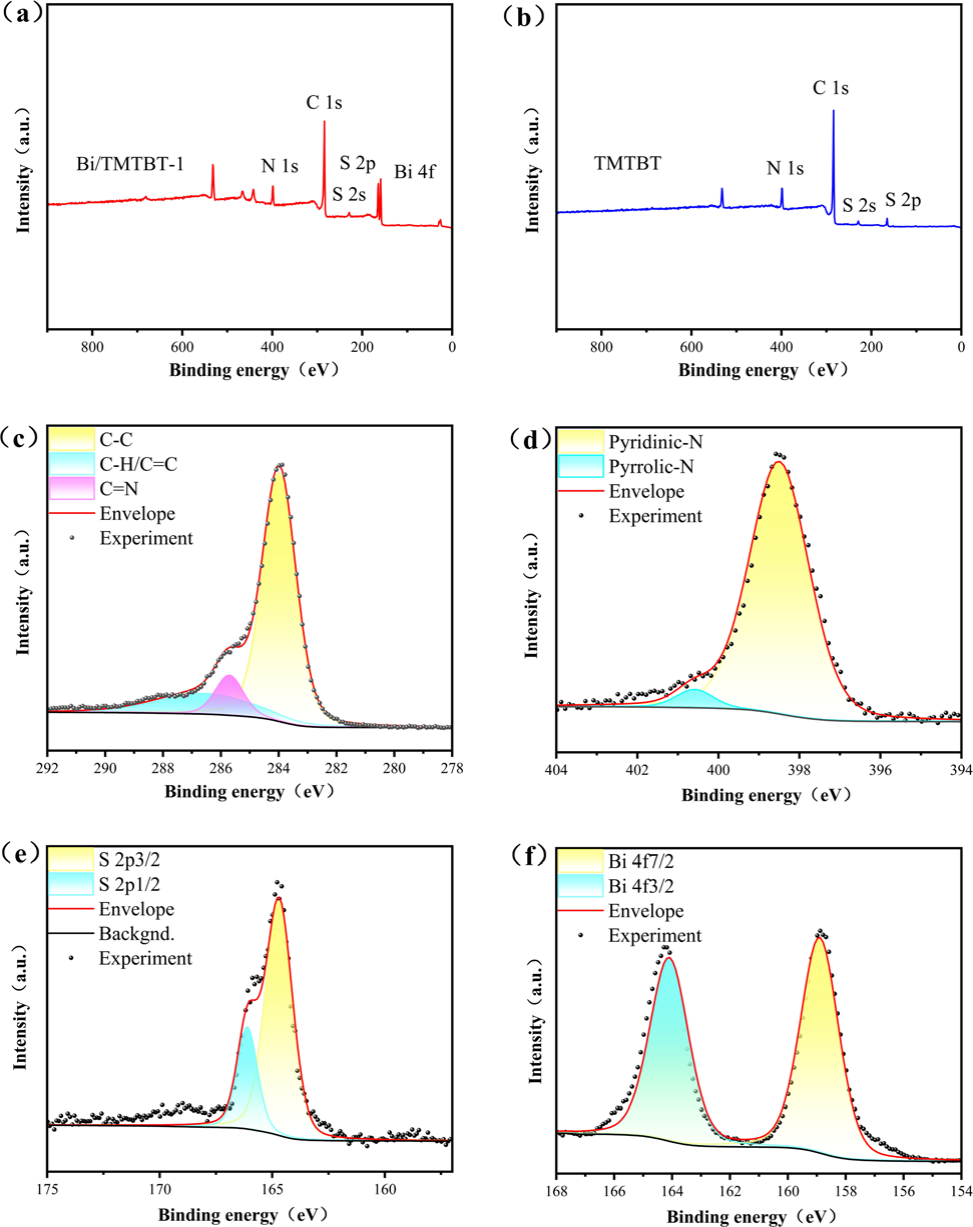
(a) XPS survey spectrum of Bi/TMTBT-1 and (b) TMTBT, and high-resolution XPS spectra of (c) C 1s, (d) N 1s, (e) S 2p, and (f) Bi 4f of Bi/TMTBT-1.

Fig. 3a and b presents the X-ray photoelectron spectroscopy (XPS) analysis of TMTBT and Bi/TMTBT-1. The C 1s spectrum of the Bi/TMTBT-1 sample (Fig. 3c) exhibits three distinct peaks at 283.98 eV, 285.70 eV, and 286.50 eV, which can be attributed to C–C, C–H, and C=N bonds, respectively.^28,29^ In Fig. 3d, the N 1s spectrum reveals two peaks at 398.50 eV and 400.58 eV, corresponding to pyridine-N and pyrrole-N in Bi/TMTBT-1.^30–32^ The high-resolution S 2p XPS spectrum, as shown in Fig. 3e, displays a peak at 164.70 eV with a shoulder peak at 166.10 eV, assigned to benzothiadiazole S 2p3/2 and S 2p1/2.^33^ In Fig. 3f, peaks at 164.10 eV and 158.90 eV correspond to Bi 4f5/2 and Bi 4f7/2, respectively.^34^ These results further confirm the successful preparation of TMTBT.

**Fig. 4:**
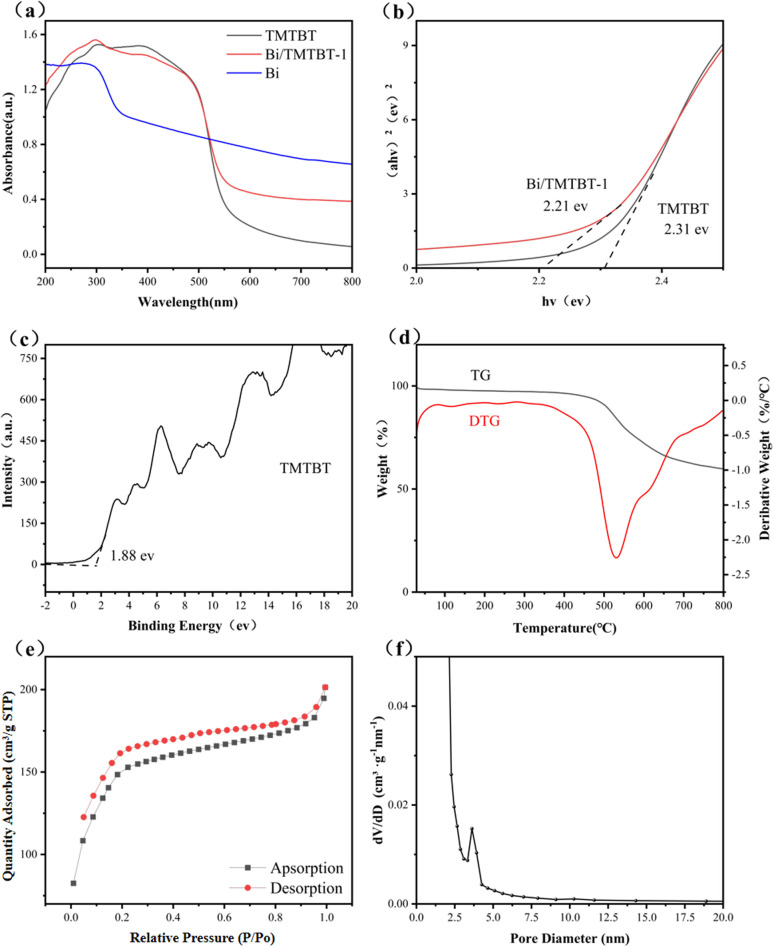
(a) UV–vis diffuse reflectance spectra of TMTBT, Bi/TMTBT-1, and Bi. (b) Plots of (*ahv*)^2^*versus* photon energy (*hv*) for TMTBT and Bi/TMTBT-1 with the corresponding band gap energies. (c) VB-XPS spectra of TMTBT. (d) Thermogravimetric analysis (TGA) of TMTBT. (e) Nitrogen adsorption–desorption isotherm of TMTBT and (f) the corresponding pore-size distribution of TMTBT.

The solid UV-Vis diffuse reflectance spectra (DRS) of all the prepared samples are depicted in Fig. 4a. Firstly, TMTBT and Bi/TMTBT-1 exhibit extensive light absorption within the UV-visible region. The UV-Vis DRS exhibited the typical LSPR absorption profile. Compared with pure TMTBT-CPP, the Bi-modified composite exhibits a distinct broad absorption enhancement in the visible-near-infrared region (550–800 nm), which is a typical optical feature originating from the LSPR effect of metallic Bi nanoparticles. This enhanced light absorption, derived from the Bi LSPR effect, effectively improves photon utilization, generates abundant hot electrons, and promotes the separation and transfer of photogenerated charges, thereby significantly boosting the photocatalytic performance. Secondly, according to the solid UV-Vis DRS, both TMTBT and Bi/TMTBT undergo direct transitions, as described by the equation *ahv* = *A*(*hv* − *E*g)^*n*/2^, where *n* is equal to 1. Furthermore, Fig. 4b shows a plot of (*ahv*)^2^ against *hv*, indicating that, after complexation, the bandgap is reduced and the visible-light absorption is enhanced. The band energy values of TMTBT and Bi/TMTBT-1 were calculated to be 2.31 eV and 2.21 eV, respectively. Moreover, the valence band (VB) position of TMTBT was measured *via* VB-XPS spectroscopy, as presented in Fig. 4c. The VB value of TMTBT is 1.88 V. By combining this with the Eg values, the corresponding conduction band (CB) positions of TMTBT were calculated to be −0.43 eV. Thermogravimetric analysis (TGA) demonstrates that TMTBT exhibits high thermal stability, with a weight loss of less than 10% at temperatures above 500 °C (decomposition commences at 470 °C). The peak weight loss rate occurs at approximately 530 °C, as indicated by the derivative thermogravimetric (DTG) curve (Fig. 4d). Moreover, the porosity and specific surface area (BET) of TMTBT were evaluated *via* nitrogen adsorption–desorption isotherms at 77 K. As shown in Fig. 4e, TMTBT exhibits a Type II isotherm, with a specific surface area of 569.16 m^2^ g^−1^, significantly higher than the previously reported value of 342.5 m^2^ g^−1^.^25^ Apart from mesopores, the abundant micropore structure also plays a crucial role in achieving such a high specific surface area. The pore diameter is 3.76 nm (Fig. 4f), consistent with findings in the literature.

**Fig. 5:**
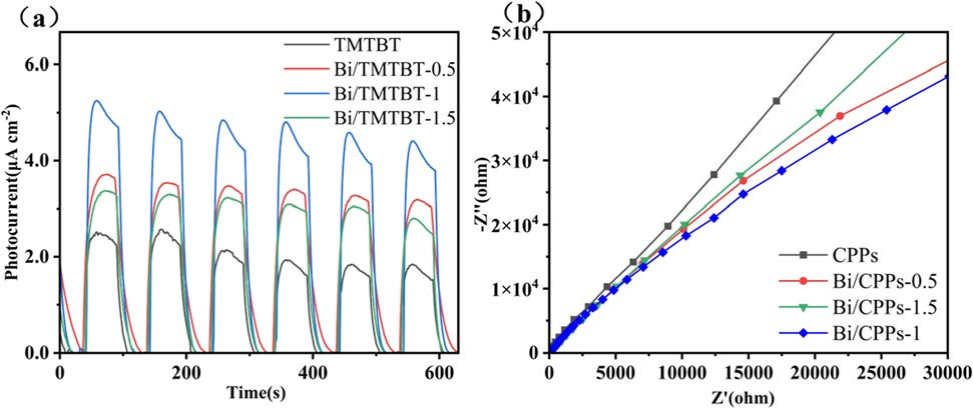
(a) Transient photocurrent response curves and (b) EIS spectra of samples under visible light.

The separation and recombination capabilities of electron–hole charges in photocatalysts were examined *via* photoelectrochemical current response and electrochemical impedance spectroscopy. As depicted in Fig. 5a, all eligible samples displayed stable photoelectrochemical current cycles under intermittent visible-light illumination, with six on–off cycles. In comparison to TMTBT, the Bi/TMTBT-1 sample presented an excellent photoelectrochemical current response. These findings imply that the Bi/TMTBT-1 heterojunction possesses a higher separation efficiency of electron–hole pairs than its individual constituents.^34^ Moreover, Fig. 5b shows the EIS variations of all samples. Generally, a smaller arc radius in the Nyquist plot represents lower charge-transfer resistance. The arc radius of Bi/TMTBT-1 is smaller than that of other materials, suggesting the most rapid charge transfer. As a result, after recombination, the photocatalytic activity is notably higher than that of TMTBT.

**Fig. 6:**
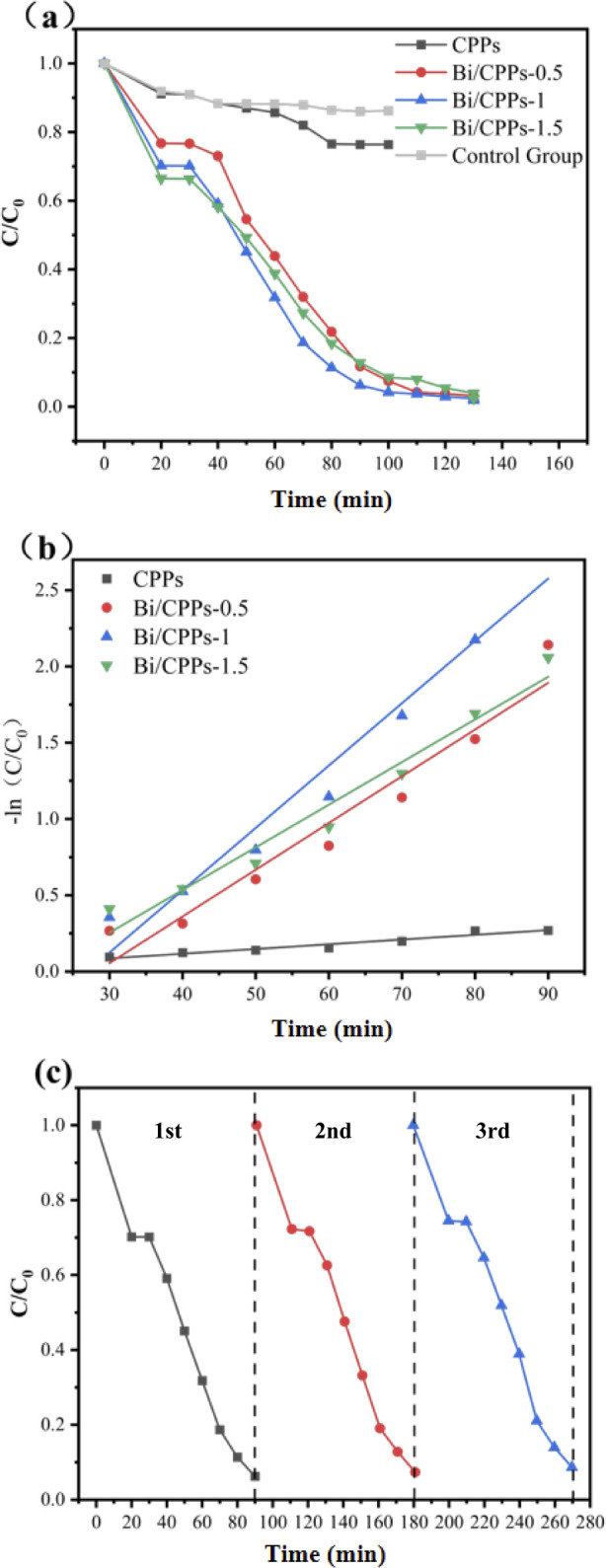
(a) Photocatalytic degradation of RhB. (b) Photocatalytic degradation kinetics of RhB by TMTBT, Bi/TMTBT-*x*, and the control group. (c) RhB degradation activity within three cycles of the Bi/TMTBT-1 photocatalyst.

As shown in Fig. 6a and b, the photocatalytic degradation performance of Bi/TMTBT-*x* composite materials was investigated. Five milligrams of TMTBT or Bi/TMTBT were added to 50 mL of an aqueous solution with a RhB concentration of 20 mg L^−1^. After 30 minutes of stirring in the dark, the adsorption–desorption equilibrium of RhB by these photocatalysts was attained. Subsequently, the photocatalytic degradation of RhB under visible-light irradiation was explored. By monitoring the variations in the RhB concentration within the solution, it was found that the degradation performance of the Bi/TMTBT heterojunction was significantly enhanced compared to that of TMTBT. In order to verify the performance of Bi/TMMTBT, we set up a control group for experiments in the absence of light and Bi loading. The results showed that light excitation and Bi loading were important conditions for the degradation of dyes. Among the synthesized Bi/TMTBT heterojunctions with different Bi contents, the Bi/TMTBT-1 heterojunction exhibited the highest photocatalytic activity, achieving a photodegradation efficiency of 85.7% within 1 hour. The photocatalytic stability of the Bi/TMTBT-1 heterojunction was also evaluated. After three cycles of RhB degradation, the photodegradation efficiency remained at 73.2%, without obvious performance decay (Fig. 6c), demonstrating favorable recyclability of Bi/TMTBT-1. SEM and XRD characterizations of cycled Bi/TMTBT-1 (Fig. S4) reveal detachment and oxidative consumption of loaded Bi nanoparticles, which is the primary factor responsible for the slight drop in cyclic activity. Further research will focus on improving the adhesion and chemical stability of plasmonic Bi nanoparticles, especially *via* surface coordination modification with organic ligands to mitigate Bi corrosion during cycling.

**Fig. 7:**
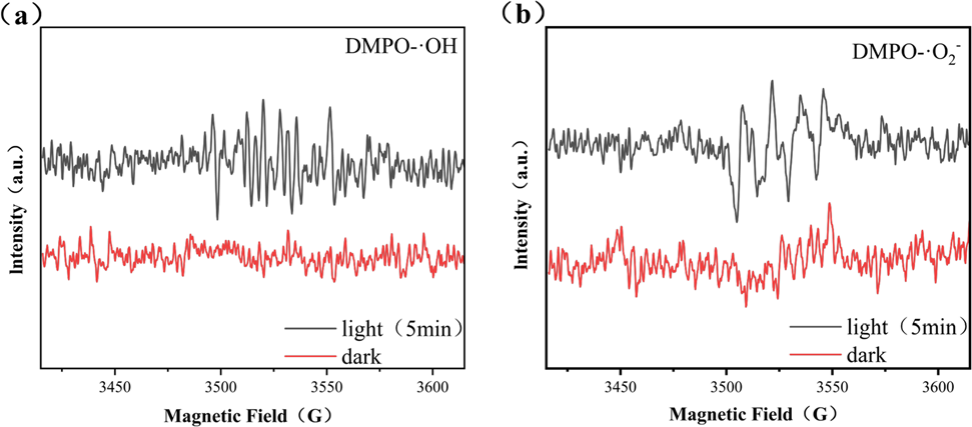
EPR spectra of (a) DMPO (5,5-dimethyl-1-pyrroline-*N*-oxide)-˙OH in aqueous solution and (b) DMPO-˙O_2_^−^ in methanol solution recorded with the Bi/TMTBT-1 sample under visible-light irradiation.

Subsequently, the active oxygen species participating in the photodegradation of RhB were identified *via* radical-trapping experiments. In this experiment, 5,5-dimethyl-1-pyrroline *N*-oxide (DMPO) was employed as a radical scavenger to stabilize the superoxide radicals (˙O_2_^−^) and hydroxyl radicals (˙OH) in methanol and aqueous solutions, respectively. As depicted in Fig. 7, no distinct signals of DMPO-˙O_2_^−^ and DMPO-˙OH were observed under dark conditions for the Bi/TMTBT-1 sample. After 5 minutes of visible-light irradiation, multiple DMPO-˙OH signals were detected, with an intensity ratio of 1 : 1 (Fig. 7a). Moreover, three characteristic peaks of DMPO-˙O_2_^−^ with an intensity ratio of 1 : 1 : 1 were clearly discerned (Fig. 7b). These findings imply that Bi/TMTBT-1 is capable of generating ˙O_2_^−^ and ˙OH radicals during the visible-light irradiation reaction process.

**Fig. 8:**
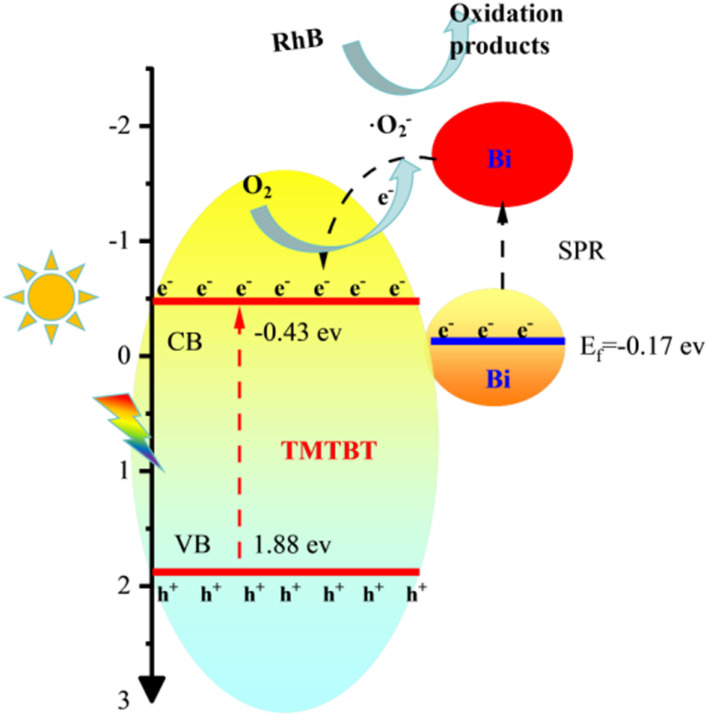
Schematic of the mechanism of Bi/TMTBT-1 under visible-light irradiation.

**Fig. 9:**
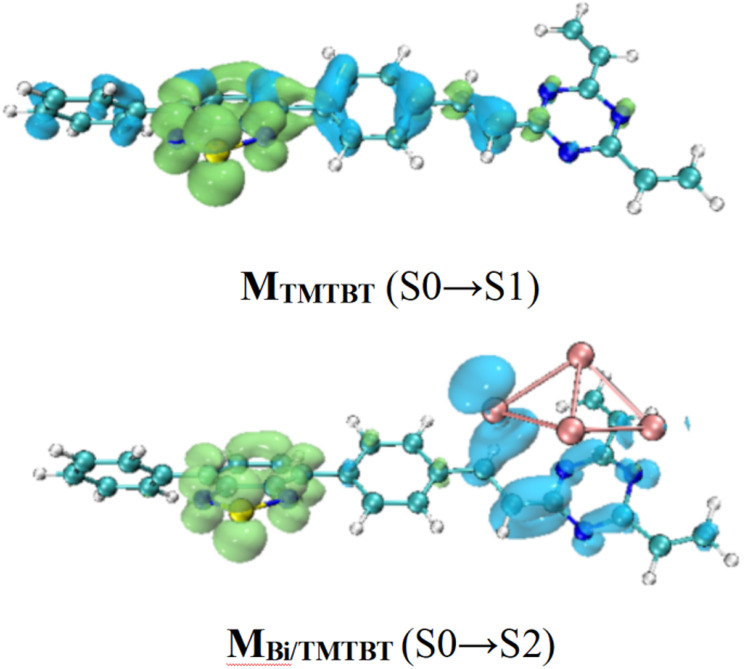
Real space representation of the hole and electron distributions of M_TMTBT_ and M_Bi/TMTBT_ (isovalue = 0.002). Green and blue regions denote the electron and hole distributions, respectively.

Based on the above analysis and results, this work puts forward a potential photocatalytic mechanism for Bi/TMTBT (Fig. 8) and the reaction process eqn (1)–(5). When TMTBT forms a heterojunction with Bi, an internal electric field is established, which promotes electron transfer. Upon exposure to visible-light irradiation, electrons are excited from the valence band (VB) of TMTBT to its conduction band (CB). Considering that the Fermi level Ef of Bi (−0.17 eV) is lower than that of the CB (−0.43 eV), electrons migrate from the CB of TMTBT to Bi.^35^ However, under visible-light illumination, the thermal electrons generated by Bi after photon absorption are elevated from the state of thermal equilibrium to high-energy states above Bi’s Fermi level.^36^ Eventually, these thermal electrons cross the heterojunction from Bi to the CB of TMTBT. To gain insight into the photophysical properties of the investigated complexes, density functional theory (DFT) calculations were performed (see more details in the SI). As shown in Fig. 9, TMTBT exhibits intramolecular charge transfer (ICT) in the absence of Bi, accompanied by inefficient separation of electrons and holes. With Bi introduced, electrons can be injected from Bi into TMTBT, and the separation of electrons and holes is significantly improved.

1 Bi/TMTBT + *hυ* → e^−^ + h^+^

2 O_2_ + e^−^ → ˙O_2_^−^

3 O_2_ + 2e^−^ + H_2_O → H_2_O_2_ + OH^−^

4 H_2_O_2_ + e^−^ → ˙OH + OH^−^

5 H_2_O + 2h^+^ → 1/2O_2_ + 2H^+^

Since the CB (−0.43 eV) has a more negative potential than that of the O_2_/˙O_2_^−^ redox couple (−0.33 eV), the electrons in the CB can reduce O_2_ to ˙O_2_^−^ radicals. In addition, the surface plasmon resonance (SPR) effect of Bi generates an electromagnetic field, which enhances electron transfer and reduces the recombination of electron–hole pairs.^37^ As a result, the lifetime of the holes in the VB of TMTBT is extended, which is conducive to the oxidative degradation of organic dyes, given that the oxidation–reduction potential of the O_2_/H_2_O_2_ couple is 0.695 eV. Therefore, the signal of ˙OH detected by EPR can be ascribed to eqn (3) and (4).^38–40^

## 4 Conclusions

In summary, a high-stability vinylene-linked TMTBT-CPP supported Bi plasmonic heterojunction photocatalyst was successfully prepared *via* an *in situ* growth strategy. Benefiting from its larger specific surface area and better thermal stability than homologous COF materials, TMTBT-CPP provides abundant active sites and a stable reaction interface, enabling the uniform immobilization of Bi nanoparticles. The optimized Bi/TMTBT composite exhibits excellent visible-light photocatalytic performance for RhB degradation and favorable cycling stability under neutral conditions. Combined with experimental results and DFT theoretical calculations, the enhanced photocatalytic mechanism was clearly elucidated. The heterojunction-induced internal electric field and Bi-based SPR effect synergistically accelerate interfacial electron migration, effectively suppress charge recombination and prolong carrier lifetime. Thermodynamically, the suitable band structure endows the composite with a strong ability to generate ˙O_2_^−^ and ˙OH active radicals, thereby realizing efficient organic pollutant degradation. This work clarifies the synergistic effect between CPP D-A structural advantages and Bi plasmonic enhancement, offering a feasible strategy for the design and preparation of high-efficiency organic composite photocatalysts for environmental remediation.

## Author contributions

Shunzhong Gong: investigation, data curation, software, formal analysis, visualization, writing – original draft, writing – review and editing; Shan Jiang: investigation, validation, visualization, partial writing – original draft, writing – review and editing; Kong Liu: conceptualization, methodology, resources, funding acquisition, project administration, writing – original draft, writing – review and editing; Jin Bai: investigation; Rao Tao: supervision; Yepeng Yang: supervision; Shulin Gao: investigation and DFT simulations; Yeit Haan Teow: writing – review and editing; Haidong Ju: conceptualization, methodology, resources, funding acquisition, project administration. All authors have read and agreed to the published version of the manuscript.

## Conflicts of interest

There are no conflicts to declare.

## Supplementary Material

RA-OLF-D6RA03144G-s001

## Data Availability

The data related to the research are included in this article. For further inquiries, please contact the corresponding author directly. All results pertinent to this study are included in the main text. Supplementary information: theoretical calculation details, as well as BET, XRD and SEM characterizations of Bi-loaded composites. See DOI: https://doi.org/10.1039/d6ra03144g.
