# Supplementary material for: Construction of a hierarchically porous A–π–A conjugated porous polymer (CPP)/Bi nanoparticle heterojunction with enhanced photocatalytic performance
Source: RSC Adv. 2026 Jul 6;16(35):37036–43. doi: 10.1039/d6ra03144g (PMC13334357; doi:10.1039/d6ra03144g)
Supplement: RA-016-D6RA03144G-s001 [file RA-016-D6RA03144G-s001.pdf]

Supplementary Information (SI) for RSC Advances.
This journal is © The Royal Society of Chemistry 2026

Supplementary Information

Construction of Hierarchically Porous A-π-A Conjugated Porous Polymer (CPP)/Bi Nanoparticles Heterojunction with Enhanced Photocatalytic Performance

Shunzhong Gong[1]†, Jiang Shan[1]†, Kong Liu[1*], Jin Bai[1], Rao Tao[1], Yepeng Yang[1], Shulin Gao[1], Yeit Haan Teow[2], Haidong Ju[1*]

Affiliations:

[1]Yunnan Key Laboratory of Metal-Organic Molecular Materials and Device, Yunnan Engineering Technology Research Center for Plastic Films, School of Chemistry and Chemical Engineering. Kunming University, Kunming 650214, PR China

[2]Department of Chemical and Process Engineering, Faculty of Engineering & Built Environment, Universiti Kebangsaan Malaysia, 43600 UKM Bangi, Selangor Darul Ehsan, Malaysia.

[*]Corresponding author: skyleo2003@126.com(K. L.) and hdju1977@outlook.com (H. J.)

†These two authors contributed equally to this work.

Table of Contents

1. Computational details

2. BET, SEM and XRD for the recycled sample after photocatalysis test of CPP supported nano Bi

1. Computational details

Kohn-Sham density functional theory (DFT) has been employed to optimize the ground state geometries of the investigated complexes at the PBE0-D3(BJ)[1, 2]/def2-SVP level. All the optimized geometries were tested to be local minima by frequency calculations at the same level. To get insight into the photophysical properties of the investigated complexes, time-dependent density functional theory (TD-DFT) calculations at the PBE0-D3(BJ)/def2-TZVP have been performed. The effect of the solvent was considered in all DFT and TD-DFT calculations utilizing the integral equation formalism polarized continuum model (IEF-PCM)[3, 4] with water as solvent. All the DFT and TD-DFT calculations were performed using the Gaussian 16 software suit.[5]

The computational models of Bi nanoparticles ($\mathbf{M_{Bi}}$), tri(methylthio)triazine and benzothiadiazole ($\mathbf{M_{TMTBT}}$) and their complex ($\mathbf{M_{Bi/TMTBT}}$) are shown in Figure S1. The choice of the computational model strikes a balance between accuracy and computational cost. As can be seen from Figure S2, the UV–Vis absorption spectra of $\mathbf{M_{Bi}}$ and $\mathbf{M_{TMTBT}}$ agree well with the experimental results (Figure 5a).

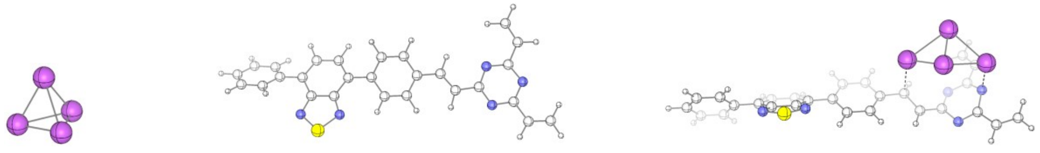

(a) (b) (c)

Fig. S1 Optimized structures for (a) $\mathbf{M_{Bi}}$; (b) $\mathbf{M_{TMTBT}}$; (c); $\mathbf{M_{Bi/TMTBT}}$

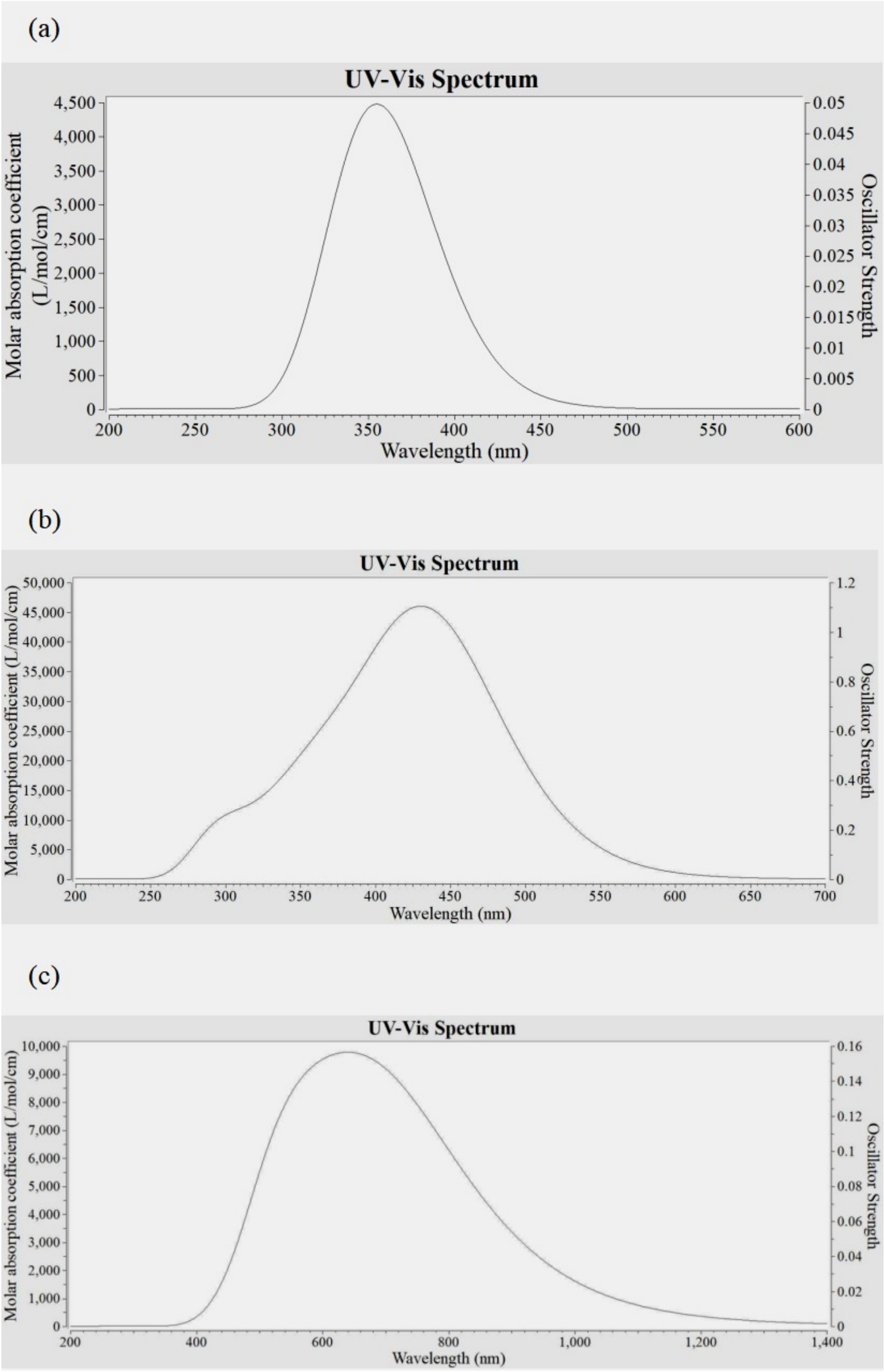

Fig. S2 Calculated Uv-Vis spectra for (a) $\mathbf{M_{Bi}}$; (b) $\mathbf{M_{TMTBT}}$; (c); $\mathbf{M_{Bi/TMTBT}}$

According to the hole-electron analysis method, the “hole” and “electron” denote where the excited electron leaves and goes, respectively. In many cases, any excitation can be identified as a definitive distribution of hole and electron. The theory proved to be a useful and powerful method in unraveling nature of electron excitations.[6] The wavefunction analysis was calculated

by means of the Multiwfn version 3.8(dev) code[7, 8] and plotted using VMD[9] and CYLview[10] software.

2. BET, SEM and XRD for the recycled sample after photocatalysis test of CPP supported nano Bi

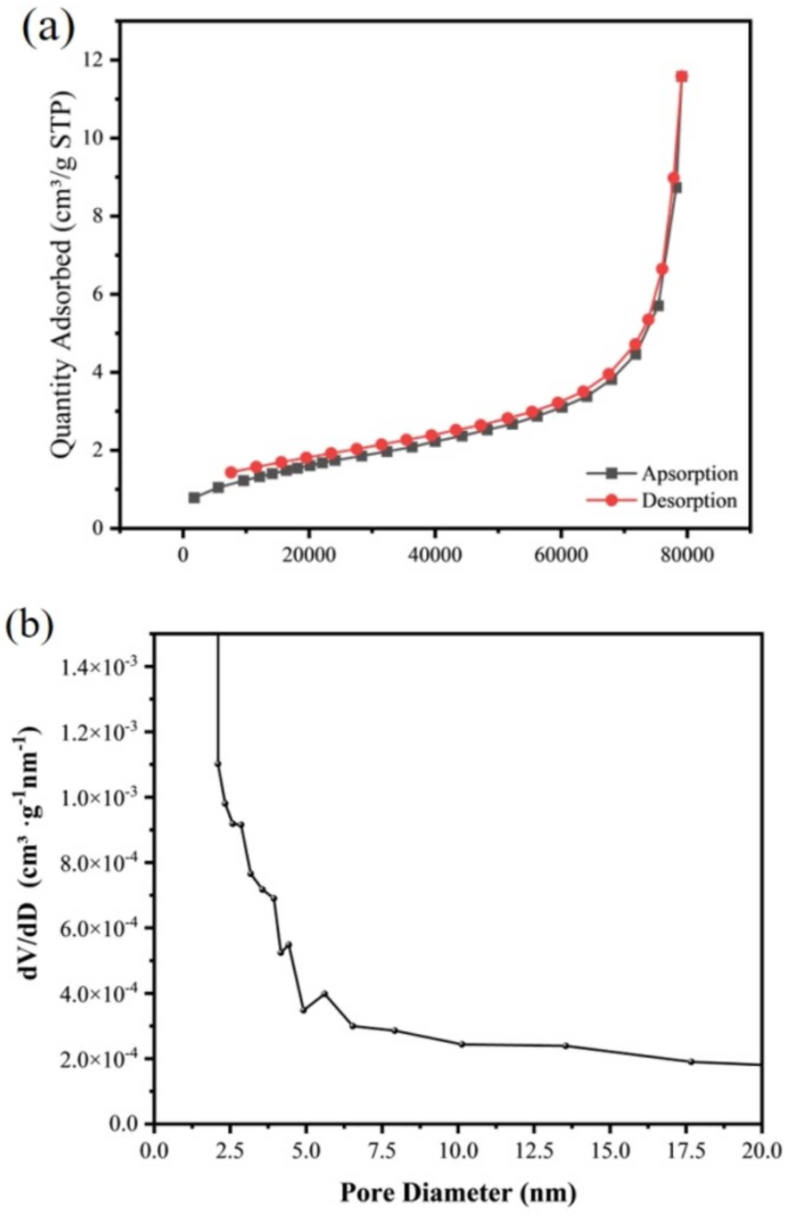

Fig. S3 BET data of Bi/TMTBT-1 after 3 cycles

As shown in the Fig. S3 is (a) Bi/TMTBT-1 nitrogen adsorption desorption isotherms, and (b) corresponding Bi/TMTBT-1 pore size distribution. After 1:1 loading of Bi, the specific surface

area of the material decreased significantly, but the number of micropores was maintained, as shown in Fig. S3 b.

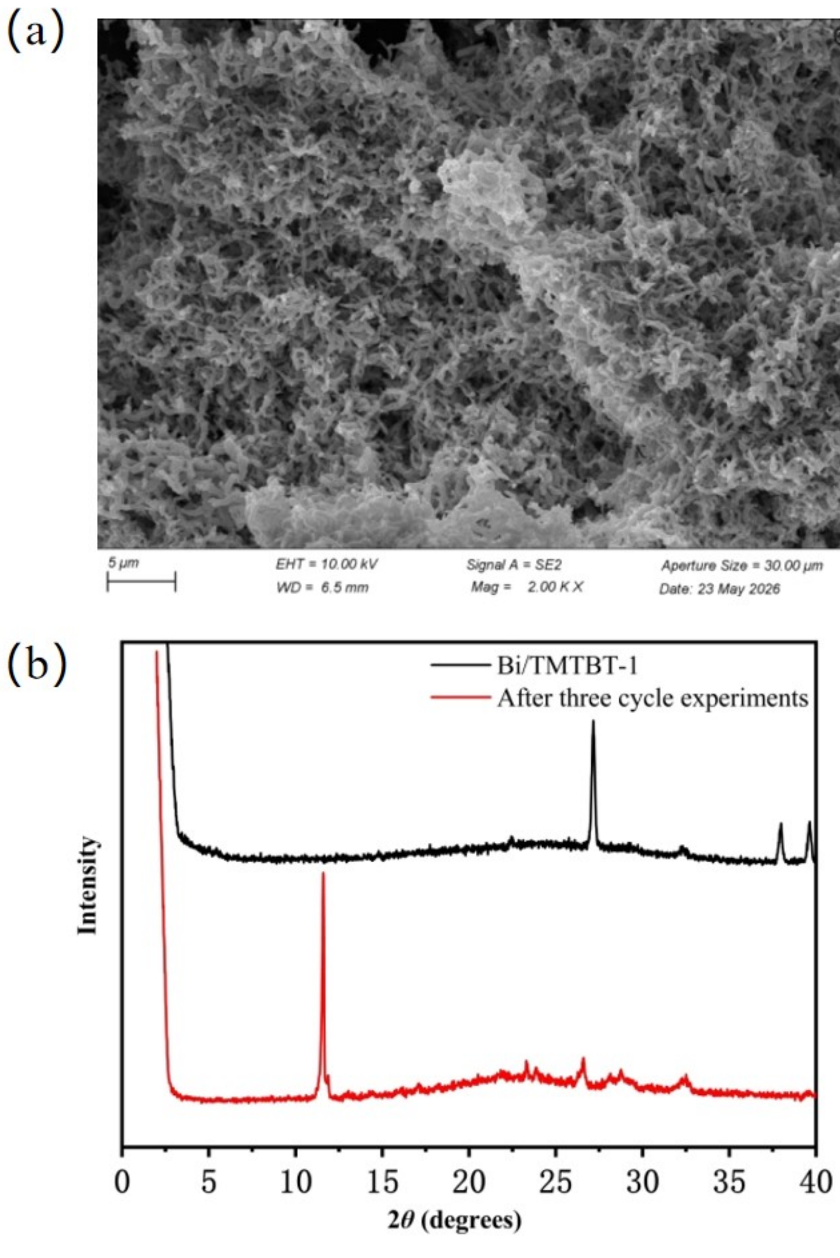

Fig. S4 SEM image and XRD data of Bi/TMTBT-1 after 3 cycles

Fig. S4 presents the SEM image and XRD patterns of Bi/TMTBT-1 after three cycles. The characterizations reveal partial phase transformation of Bi nanoparticles, together with their detachment and oxidative consumption during photocatalysis.

References

[1] Adamo C, Barone V. Toward reliable density functional methods without adjustable parameters: The PBE0 model. The Journal of Chemical Physics, 1999, 110: 6158-6170.

[2] Grimme S, Ehrlich S, Goerigk L. Effect of the damping function in dispersion corrected density functional theory. Journal of Computational Chemistry, 2011, 32: 1456-1465.

[3] Mennucci B, Cancès E, Tomasi J. Evaluation of solvent effects in isotropic and anisotropic dielectrics and in ionic solutions with a unified integral equation method: theoretical bases, computational implementation, and numerical applications. The Journal of Physical Chemistry B, 1997, 101: 10506-10517.

[4] Cancès E, Mennucci B, Tomasi J. A new integral equation formalism for the polarizable continuum model: theoretical background and applications to isotropic and anisotropic dielectrics. The Journal of Chemical Physics, 1997, 107: 3032-3041.

[5] Frisch M J, Trucks G W, Schlegel H B, et al. Gaussian 16 Rev. A.03. Gaussian Inc, 2016.

[6] Liu Z, Lu T, Chen Q. An sp-hybridized all-carboatomic ring, cyclo[18]carbon: electronic structure, electronic spectrum, and optical nonlinearity. Carbon, 2020, 165: 461-467.

[7] Zhang J, Lu T. Efficient evaluation of electrostatic potential with computerized optimized code. Physical Chemistry Chemical Physics, 2021, 23: 20323-20328.

[8] Lu T, Chen F. Multiwfn: a multifunctional wavefunction analyzer. Journal of Computational Chemistry, 2012, 33: 580-592.

[9] Humphrey W, Dalke A, Schulten K. VMD: visual molecular dynamics. Journal of Molecular Graphics, 1996, 14: 33-38.

[10] Legault C Y. CYLview20. Université de Sherbrooke, 2020.
